# Seasonal changes in leaf chemistry and leaf selection of the Japanese giant flying squirrel upon two tree species

**DOI:** 10.1002/ece3.3155

**Published:** 2017-06-15

**Authors:** Mutsumi Ito, Noriko Tamura, Fumio Hayashi

**Affiliations:** ^1^ Department of Biology Tokyo Metropolitan University Hachioji Tokyo Japan; ^2^ Tama Forest Science Garden Forestry and Forest Products Research Institute Hachioji Tokyo Japan

**Keywords:** folivory, food selection, glucose content, leaf‐folding, total phenolic content

## Abstract

Tree leaves are important food sources for arboreal herbivores, such as primates, rodents, and marsupials. These animals do not eat leaves randomly in habitats with many tree species but rather choose based on the chemical components of leaves, such as sugars, fibers, proteins, and toxins. However, the effects of the microscale distribution of these chemicals within each leaf have not been examined for these animals. The giant flying squirrels *Petaurista leucogenys* are entirely arboreal, nocturnal herbivores, usually feeding on leaves and dropping leaf debris on the ground after partially consuming them. Therefore, we could easily assess which species of trees and which parts of the individual leaves they preferred to eat. We also examined microscale distributions of phenolics, sugar, and water within individual leaves. Of the two dominant food tree species, the deciduous *Quercus acutissima* was preferred over the evergreen *Q. sessilifolia*. The latter tree is only used during winter to early spring when the former had no leaves. Our chemical analyses revealed that *Q. acutissima* contained much more glucose than *Q. sessilifolia* in all seasons. Three types of leaf debris, eaten apically, basally, or centrally with a hole, were found. In *Q. sessilifolia*, which had low phenolic concentrations, apical eating was most common, whereas central eating was rare. In *Q. acutissima*, which had high phenolics, basal or central eating was common. Central feeding may be caused by avoiding the periphery because of a higher phenolic concentration in the leaf margin. Thus, microscale distributions of phenolics within individual leaves affect which parts *P. leucogenys* eats, whereas leaf sugar concentration is an important factor affecting which species of leaves they eat.

## 1 INTRODUCTION

Herbivorous mammals are forced to make optimal choices regarding their daily diets by selecting plants with higher nutritional value and by avoiding those with possible toxins to maintain and develop their bodies (Farmer, 2014; Glander, 1982). The main nutritional resources from plants are sugars, proteins, and minerals; some species have higher concentrations of these than others (Farmer, 2014). However, plants usually include fibers (e.g., cellulose, lignin, and hemicellulose), which are poor energy sources, and also develop secondary metabolites, such as toxins (e.g., phenolics and alkaloids) and digestion inhibitors (condensed tannins) (Farmer, 2014; Glander, 1982; Harborne, 1991). Food selection by herbivorous mammals based on the abundance of valuable nutrients and plant defensive chemicals has been well documented and, in most cases, they select food materials with substantial amounts of preferable nutrients (Jensen, Wallis, & Foley, 2015; Kar‐Gupta & Kumar, 1994; Matsuda, Tuuga, Bernard, Sugau, & Hanya, 2013; Remis, 2002; Remis & Kerr, 2002; Willig & Lacher, 1991). Many mammals are considered to have four primary taste modalities (sweet, salty, bitter, and sour), with umami, a savory taste associated with specific amino acids, being a fifth modality, as in humans (Lemon, 2015; Yarmolinsky, Zuker, & Ryba, 2009). Sweet and umami promote feeding behaviors, whereas bitter and sour tastes reduce food intake, and the perceptions of sweetness may contribute to preferences for fruits, flowers, and other foods high in soluble sugars (Yarmolinsky et al., 2009). Clear preferences for sweetness and/or soluble sugar have been documented in animals ranging from relatively small mammals, such as frugivorous bats (Ayala‐Berdon et al., 2011; Law, 1993), to large mammals, such as roe deer and primates (Remis, 2002; Remis & Kerr, 2002; Tixier et al., 1997). In contrast, a significant number of plant metabolites are bitter or otherwise unpleasant in taste (Harborne, 1991). The taste of bitterness may predict the deterrent effects of toxins and digestibility‐reducing tannin compounds in fruits and leaves (Critchtey & Rolls, 1996) and may be a principal cause of food rejection (Remis, Dierenfeld, Mowry, & Carroll, 2001; Takemoto, 2003). Therefore, mammalian herbivores often show behavioral avoidance and/or tolerance of plant secondary metabolites, such as high concentrations of tannins and related polyphenolics (Glander, 1982; Iason & Villalba, 2006; Sorensen, McLister, & Dearing, 2005).

Tree leaves are important foods for arboreal herbivores, such as primates, rodents, and marsupials (Farmer, 2014). The Japanese giant flying squirrel (*Petaurista leucogenys* Temminck, 1827) is an exclusively arboreal, nocturnal, and tree leaf‐eating mammal (Ohdachi, Ishibashi, Iwasa, Fukui, & Saitoh, 2015). Its home range size is 0.4–5.2 ha, usually larger in males, with considerable overlaps between the sexes and between males (Baba, Doi, & Ono, 1982; Kawamichi, 1984b). Mature *P. leucogenys* reaches weights of up to 1.3 kg (Ohdachi et al., 2015). Such large bodies may be maintained by nutrition from specialized cecal microbiota, which are known to convert diverse plant materials into absorbable nutrients in *P. alborufus lena* of Taiwan (Lu et al., 2012). *P. leucogenys* usually eats leaves from a variety of tree species in the seasonal environment of Japan and chooses different food items (Ando, Shiraishi, & Uchida, 1985; Baba et al., 1982; Kawamichi, 1997, 2010). However, the effect of leaf chemicals on food choice has not been examined. We predict that *P. leucogenys* will prefer leaves with higher concentrations of soluble sugars and will avoid leaves with higher concentrations of phenolics. To examine this hypothesis, the leaf chemicals and nutritional conditions of two dominant *Quercus* tree species, deciduous *Quercus acutissima* and evergreen *Q. sessilifolia*, were assessed seasonally in relation to food choice by *P. leucogenys*.

## 2 MATERIALS AND METHODS

### 2.1 Study site and sample collection

The single census route (2 km long, 5 m wide) was set in a 50‐ha wooded area in the Tama Forest Science Garden at Todori, Hachioji, Tokyo, Japan (35°38′50.74″N, 139°16′38.15″E). The vegetation was mixed temperate broadleaf and coniferous trees (Tamura, 2004). The climate is characterized by four seasons: spring (March to May), summer (June to August), autumn (September to November), and winter (December to February). Normal value average temperatures from 1981 to 2010 at Hachioji meteorological station are 3.2°C in January, 4.1°C in February, 7.5°C in March, 13.1°C in April, 17.6°C in May, 20.9°C in June, 24.7°C in July, 26.1°C in August, 22.2°C in September, 16.4°C in October, 10.7°C in November, and 5.7°C in December (Japan Meteorological Agency, 2017). Monthly rainfalls are 48.3 mm in January, 49.4 mm in February, 103.4 mm in March, 118.2 mm in April, 121.5 mm in May, 167.9 mm in June, 176.0 mm in July, 242.2 mm in August, 256.7 mm in September, 187.7 mm in October, 88.8 mm in November, and 45.2 mm in December (Japan Meteorological Agency, 2017).

**Figure 1:**
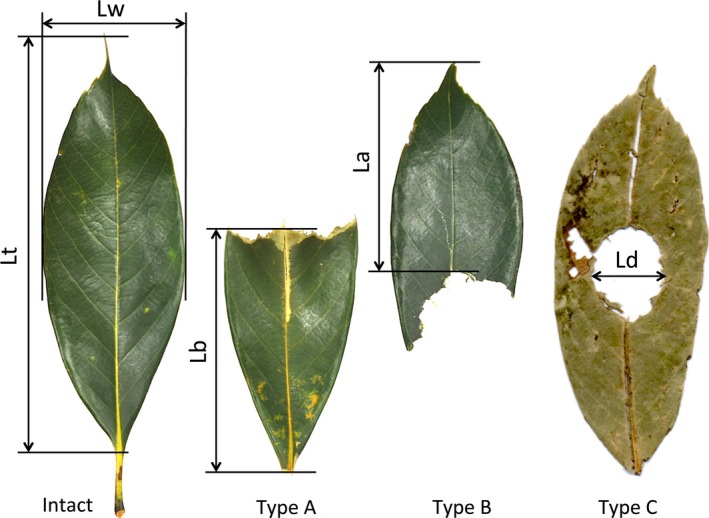
Three types of leaf debris eaten by the Japanese giant flying squirrel (Type A, apically eaten; Type B, basally eaten; Type C, only centrally eaten). The total length (Lt) and width (Lw) of intact leaves are measured, as well as the remaining length for basally (La) and apically (Lb) eaten leaves, and the maximum width of the centrally eaten circle (Ld) of leaf debris. All leaves shown are the evergreen *Quercus sessilifolia*

The morning census was conducted usually from 9 a.m. along this route, walking slowly for about 1.5 hr. It was conducted one to 5 days per month from May 2013 to November 2015, resulting a total of 87 censuses. Along the route, we collected and counted all leaf debris of *Q. acutissima* and *Q. sessilifolia* that had been eaten by *P. leucogenys*. Direct observations of feeding are necessary to demonstrate their foods (e.g., Kawamichi, 1997; Koli, Bhatnagar, & Sharma, 2013). Although we did not observe directly their feeding at night, it has been well documented for *P. leucogenys* to remain the characteristic leaf debris when they eat tree leaves (Kawamichi, 1984a, 2010; Okazaki, 2004; Shigeta & Okazaki, 2001; Shigeta, Shigeta, & Tamura, 2010). In this study therefore we used the number of such characteristic leaf debris as the feeding intensity of *P. leucogenys* upon the two tree species. *P. leucogenys* was the only mammal eating tree leaves around the census route (Mikuriya, 1993). Collected leaves were classified into three types: eaten at the apical part (Type A), eaten at the basal part (Type B), and eaten at the central part (Type C) (Figure 1). The remaining lengths of basally and apically eaten leaves (La and Lb, respectively, in Figure 1) were measured with a slide calliper to the nearest 0.1 mm. In Type C leaves, the maximum width of the hole was also measured (Ld in Figure 1). We collected several intact leaves during the censuses and measured the length excluding the petiole (Lt) and the width at the widest part (Lw) as controls (Figure 1).

### 2.2 Total phenolic content

Fresh leaves of *Q. acutissima* and *Q. sessilifolia* were collected from the branches at the study site on 15 and 17 occasions, respectively, from May 2013 to October 2015. These leaves were put in an ice box for 1 hr during transport and then stored at −30°C in a laboratory freezer. The Folin–Ciocalteau method was used to measure the total concentration of phenolic compounds in the leaves (Nurmi, Ossipov, Haukioja, & Pihlaja, 1996). Two circular disks, 17–20 mm in diameter, were cut with a cork borer at the apical and basal parts from five fully expanded and undamaged leaves. In another five leaves collected during eight samplings of *Q. acutissima* and eight samplings of *Q. sessilifolia*, the circular disk at the central part and the marginal part of both the right and left sides were cut. These leaf sections were dried at 60°C overnight and then weighed to the nearest 0.1 mg. Each dried leaf section was powdered in a 2.0‐ml tube using a pestle and mixed with 1 ml 70% aqueous acetone. The homogenate was sonicated for 10 min and centrifuged at 2,500 g for 10 min. The supernatant was stored in a 15‐ml tube. This procedure was repeated three times, and the entire 3‐ml aqueous acetone extract was fully evaporated under low pressure at 40°C. The residue was dissolved in 1 ml distilled water. After being diluted 60 times, 0.3 ml of the solution was mixed with 0.3 ml of 2N Folin reagent (Folin–Ciocalteau's phenol reagent) in a 2.0‐ml tube, allowed to stand for 5 min, and then mixed with 0.6 ml 20% Na_2_CO_3_. After a 10‐min incubation at room temperature, the mixture was centrifuged at 1,500 g for 8 min, and the absorbance at 730 nm was measured using a spectrophotometer (DU 640; Beckman Instruments, Fullerton, CA, USA). The standard curve was prepared with six concentrations of 0‐ to 50‐mg/L gallic acid, and the phenolic contents were expressed as gallic acid equivalent.

### 2.3 Sugar and water contents

We collected leaf samples six times for deciduous *Q. acutissima* and eight times for evergreen *Q. sessilifolia* from May 2015 to March 2016 to measure the sugar and water contents of leaves. All sampling was performed at the study site from 10:30 to 11:00 on clear days. After 1 hr of transport on ice to the laboratory, the leaves were cut as described for the measurement of total phenolic contents. The fresh weight of each leaf section was measured to the nearest 0.1 mg immediately after cutting, and the sections were put into 2‐ml tubes with 0.5‐ml distilled water. After being heated at 90°C for 20 min, the leaf sections were pulverized using a homogeniser. The tip of the homogeniser was washed with 0.5 ml distilled water, which was added to the same tube. The mixture was vortexed and then centrifuged at 1,500 g for 10 min, after which the supernatant was transferred into a new tube. Next, 10 μl of the supernatant was used to measure the glucose concentration with a glucose‐measuring instrument (Glutest Every, Sanwa Kagaku Kenkyusho Co., Ltd., Nagoya, Japan). The precipitate in the tube was dried at 80°C for 1 day to measure the dry weight.

### 2.4 Statistical analyses

The values were shown as the mean ± standard deviation (*SD*). Paired *t* tests were used to test for differences in the contents of total phenolics, glucose, or water between the apical and basal parts of leaves and between the central and marginal parts of leaves in each tree species. When assessing differences in leaf size and chemical traits between the two tree species, we used Student's *t* tests, with Welch's approximate *t* applied instead when variances were not equal. Interspecific differences in the frequencies of the three types of leaf debris were assessed by chi‐square (χ^2^) tests.

## 3 RESULTS

### 3.1 Leaf debris

**Figure 2:**
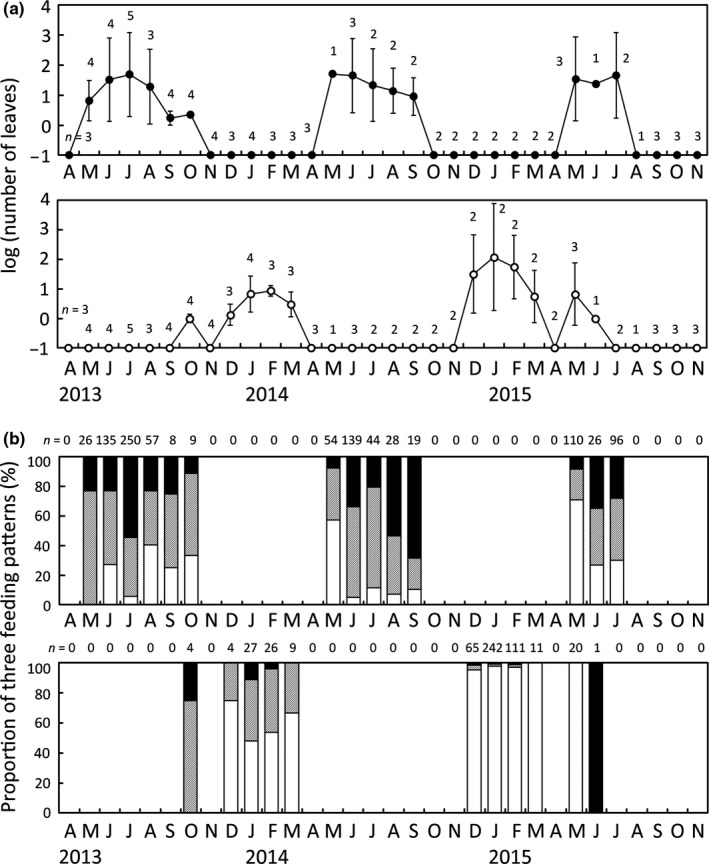
(a) The mean (± *SD*) number of the leaf debris found in each month (*n*, the number of censuses per month). When no leaf debris was observed, it was treated as 0.1 (−1 on log_10_ axis). (b) The proportions of the three types of leaf debris, Types A (white), B (shaded), and C (black), in each month (*n*, total leaf debris per month). In both (a) and (b), the upper panel shows the leaves of deciduous *Quercus acutissima,* and the lower panel shows those of evergreen *Q. sessilifolia*

Leaf debris of the deciduous *Q. acutissima* was found from May to October and was abundant in number from May to July (Figure 2a). However, we never found leaf debris of just‐expanded leaves in early May. On the other hand, leaf debris of the evergreen *Q. sessilifolia* was found from October to June and was abundant in number from December to March (Figure 2a). Of the 1,001 total feeding marks on *Q. acutissima* leaves, 240 (24.0%) were Type A, 439 (43.9%) were Type B, and 322 (32.2%) were Type C. Of the 520 total marks on *Q. sessilifolia* leaves, 474 (91.2%) were Type A, 36 (6.9%) were Type B, and 10 (1.9%) were Type C. Thus, the proportions of leaf‐feeding Types A, B, and C differed between *Q. acutissima* and *Q. sessilifolia* (χ^2^ = 621.9, *df* = 2, *p *<* *.0001). There was no clear seasonal tendency in the proportions of these three types of feeding patterns (Figure 2b).

In *Q. acutissima*, the mean Lt and Lw of intact leaves were 148.2 ± 33.2 mm (*n *=* *149) and 37.1 ± 11.5 mm (*n *=* *149), respectively. In feeding Type A, the mean Lb was 18.3 ± 20.4 mm (*n *=* *186), suggesting that 87.7% [(148.2–18.3)/148.2] of the leaf (in length) was eaten apically. In Type B, the mean La was 79.5 ± 37.3 mm (*n *=* *407), suggesting that 46.4% [(148.2–79.5)/148.2] of the leaf was eaten basally. In Type C, the mean La, Lb, and Ld were 102.4 ± 34.0 mm (*n *=* *267), 3.1 ± 7.0 mm (*n *=* *310), and 20.1 ± 5.8 mm (*n *=* *302), respectively, suggesting that the leaves were, on average, eaten more basally.

In *Q. sessilifolia*, the mean Lt and Lw of intact leaves were 102.7 ± 19.2 mm (*n *=* *69) and 30.5 ± 4.5 mm (*n *=* *69), respectively, which were lower than those of *Q. acutissima* leaves (Welch's *t*‐test: *t *=* *12.8, *df* = 206, *p *<* *.0001 in Lt; *t *=* *6.1, *df* = 211, *p *<* *.0001 in Lw). In feeding Type A, the mean Lb was 21.2 ± 16.2 mm (*n *=* *470), suggesting that 79.4% [(102.7–21.2)/102.7] of the leaf (in length) was eaten apically. In Type B, the mean La was 46.0 ± 17.0 mm (*n *=* *30), suggesting that 55.2% [(102.7–46.0)/102.7] of the leaf was eaten basally. In Type C, the mean La, Lb, and Ld were 33.2 ± 12.3 mm (*n *=* *10), 3.3 ± 10.0 mm (*n *=* *9), and 12.1 ± 6.9 mm (*n *=* *8), respectively, suggesting that the leaves were, on average, eaten more basally.

### 3.2 Leaf chemicals

**Figure 3:**
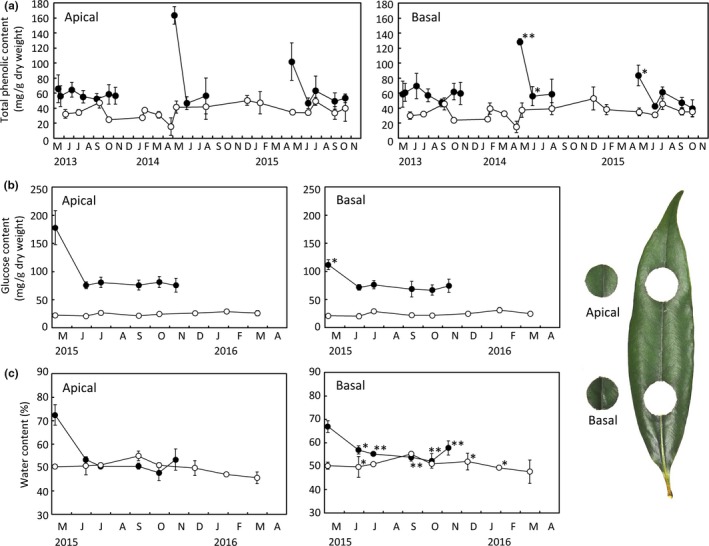
Seasonal changes in (a) the mean total phenolic content (gallic acid equivalent, ± *SD*), (b) the mean glucose content (± *SD*), and (c) the mean water content (± *SD*) in the apical and basal parts of leaves of deciduous *Quercus acutissima* (closed circles) and evergreen *Q. sessilifolia* (open circles). The number of leaves examined during each collection was *n *=* *5, excluding those collected for glucose and water contents in May (*n *=* *4). *p <* *.05 and **p <* *.01 in the paired *t* tests between the apical and basal parts

In the deciduous *Q. acutissima*, foliation occurred during April, and the fully expanded leaves were still soft and light green in early May. Total phenolic content was much higher and more condensed in the apical parts of the leaves in early May than in other months (Figure 3a). If these data in early May were excluded, the average total phenolic contents were 55.7 ± 6.2 (*n *=* *13) mg/g dry weight in apical parts and 55.4 ± 8.7 (*n *=* *13) mg/g dry weight in basal parts. In the evergreen *Q. sessilifolia*, there were no such trends, and total phenolic contents were always lower than those in *Q. acutissima* (Figure 3a); the average was 36.6 ± 9.3 (*n *=* *17) mg/g dry weight in apical parts and 34.9 ± 8.9 (*n *=* *17) mg/g dry weight in basal parts (Student's *t* tests: *t *=* *6.4, *df* = 28, *p *<* *.001 in apical parts; *t *=* *6.3, *df* = 28, *p *<* *.001 in basal parts).

In the deciduous *Q. acutissima*, the glucose contents were higher and more condensed in the apical parts of newly expanded leaves in early May (Figure 3b). In the evergreen *Q. sessilifolia*, however, such trends were not detected, and glucose was always much lower than in *Q. acutissima* (Figure 3b). In *Q. acutissima*, the average glucose contents were 78.0 ± 2.8 (*n *=* *5) mg/g dry weight in apical parts and 71.7 ± 3.9 (*n *=* *5) mg/g dry weight in basal parts (excluding the values in early May), whereas in *Q. sessilifolia*, the glucose contents were 24.8 ± 2.8 (*n *=* *8) mg/g dry weight in apical parts and 24.6 ± 3.9 (*n *=* *8) mg/g dry weight in basal parts (Student's *t* tests: *t *=* *33.2, *df* = 11, *p *<* *.0001 in apical parts; *t *=* *21.2, *df* = 11, *p *<* *.0001 in basal parts).

Newly expanded leaves of *Q. acutissima* included more water than did those in other seasons (Figure 3c). When excluding the former, water contents were higher in basal than in apical parts of leaves during any season (Figure 3c). In *Q. sessilifolia*, however, such tendencies were unclear. The lower water contents in apical parts in winter (December and January) were statistically significant (see Figure 3c), as were higher water contents in apical parts in June (Figure 3c). The average water contents of leaves were similar between the two species, although they differed slightly in the basal parts; the average was 51.1% ± 2.4 (*n *=* *5) in apical parts and 55.2% ± 2.3 (*n *=* *5) in basal parts in *Q. acutissima*, excluding the values in early May; they were 50.1% ± 2.8 (*n *=* *8) in apical parts and 50.8% ± 2.2 (*n *=* *8) in basal parts in *Q. sessilifolia* (Student's *t* tests: *t *=* *0.7, *df* = 11, *p *=* *.52 in apical parts; *t *=* *3.5, *df* = 11, *p *<* *.01 in basal parts).

**Figure 4:**
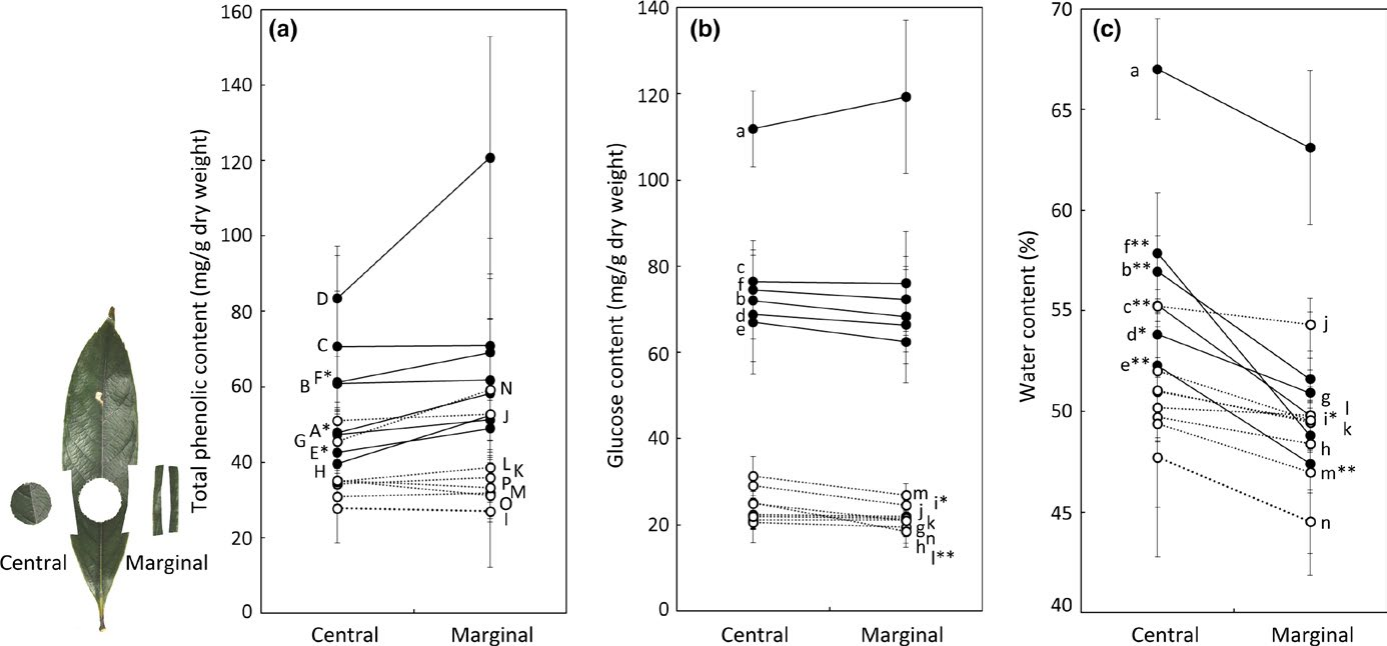
The mean contents of (a) total phenolics (gallic acid equivalent, ± *SD*), (b) glucose (± *SD*), and (c) water (± *SD*) at the central and marginal parts of leaves of deciduous *Quercus acutissima* (closed circles and labeled at left) and evergreen *Q. sessilifolia* (open circles and labeled at right). The number of leaves examined during each collection was *n *=* *5, excluding those collected for total phenolic contents on 24 June 2013 (*n *=* *10) and for glucose and water contents on 5 May 2015 (*n *=* *4). *p <* *.05 and **p <* *.01 in the paired *t* tests between the central and marginal parts of a leaf. The dates of leaf sampling for total phenolics: A, 24 June 2013; B, 11 August 2014; C, 23 August 2014; D, 2 May 2015; E, 22 June 2015; F, 15 July 2015; G, 12 September 2015; H, 14 October 2015; I, 27 January 2014; J, 3 February 2014; K, 19 March 2014; L, 5 May 2015; M, 22 June 2015; N, 15 July 2015; O, 12 September 2015; and P, 14 October 2015. Dates for glucose and water: a/g, 5 May 2015; b/h, 22 June 2015; c/i, 15 July 2015; d/j, 12 September 2015; e/k, 14 October 2015; f, 9 November 2015; l, 9 December 2015; m, 27 January 2016; and n, 15 March 2016

In *Q. acutissima*, the phenolics were always more condensed at the leaf margin than at the center, which was statistically significant in three of eight samplings (Figure 4a). In *Q. sessilifolia*, however, the phenolics were distributed homogeneously in the leaves (not significant in any sampling; Figure 4a). On the other hand, the glucose contents did not differ between the center and the margin in either *Q. acutissima* or *Q. sessilifolia* (significant in only two of eight samplings in the latter species; Figure 4b). The water contents were always higher in the central parts of leaves than at the margins, and these differences were statistically significant in five of six samplings in *Q. acutissima* and two of eight samplings in *Q. sessilifolia* (Figure 4c).

## 4 DISCUSSION

### 4.1 Food selection

*Petaurista leucogenys* preferred the leaves of deciduous *Q. acutissima* to those of evergreen *Q. sessilifolia*. Despite being available all year round, the leaves of the latter were rarely used as food when those of the former were available. Our chemical measurements revealed that *Q. acutissima* had much higher glucose contents than *Q. sessilifolia* at all time. This may be one of the reasons *P. leucogenys* preferred *Q. acutissima* leaves. In general, mammals prefer to eat sweet food materials because sweetness is a reliable marker of energy content (Laska, 1996; Lemon, 2015). Preferences by sugar composition have been also examined in primates, bats, rock mice, and possums; some have significant preferences for specific types of sugars (e.g., Johnson, van Tets, & Nicolson, 1999; Laska, 1997; Laska, Sanchez, & Luna, 1998), whereas others do not (e.g., Coleman & Downs, 2012; Landwehr, Richardson, & Wooller, 1990; Rodríguez‐Peña et al., 2007). Moreover, the previous studies have shown that primates prefer to eat plants that are high in proteins and low in fiber (Ganzhorn, 1992; Kar‐Gupta & Kumar, 1994; Norscia, Ramanamanjato, & Ganzhorn, 2012; Oates, Waterman, & Choo, 1980; Wasserman & Chapman, 2003). Thus, the effects of sugars other than glucose, proteins, and fibers on the food selection of *P. leucogenys* should be studied in the future.

The sweeter leaves of *Q. acutissima* also had much higher levels of phenolics, which animals often avoid (Farmer, 2014). However, *P. leucogenys* tended to feed on *Q. acutissima* despite the higher concentration of phenolics. This situation may be similar to fruit‐eating gorillas and other primates who tolerate higher concentrations of tannins when the latter are present in high‐sugar solutions (Remis & Kerr, 2002; Remis et al., 2001) because the perceived intensity of the tannic acid is decreased by sweetness (Lyman & Green, 1990). In most other mammals, however, the effects on food selection of sugar versus tannins and other phenolics remain unknown. It is unclear whether *P. leucogenys* is more tolerant of these chemicals as sugar concentration increases.

In this study, the selection of only the two species of *Quercus* leaves is focused. However, flying squirrels usually use a variety of food in the wild; leaves, buds, flowers, seeds, fruit, twigs, bark, pith, fungi, and lichens (review by Koli et al., 2013; and also Thysell, Villa, & Carey, 1997; Currah, Smreciu, Lehesvirta, Niemi, & Larsen, 2000; Mitchell, 2001). In the future, we will need evaluation of food availability and usage among these variable food items to understand food selectivity of *P. leucogenys*.

### 4.2 Feeding behavior

*P. leucogenys* leaves behind three types of leaf debris: those fed on apically, basally, and centrally (Types A, B, and C in Figure 1, respectively). Before eating each leaf, they often fold the leaf once, twice, or more times with their forelegs (Ito et al., 2016; Kawamichi, 1984a; Okazaki, 2004; Shigeta & Okazaki, 2001; Shigeta et al., 2010). When the leaf is folded twice (first longitudinally, then laterally) and the folded corner is eaten, the Type C marking remains on that leaf (Ito et al., 2016). Such skillful and complex feeding behavior is generally seen only in local populations because of limited cultural transmission by social learning (e.g., Laland, 2008). In *P. leucogenys*, the leaves with central eating are found in some local populations (Ito et al., 2016), and so that it may be interesting to examine how they learn it.

At our study site, they often showed such complex feeding behavior, but the proportions of the three types of leaf debris differed greatly between *Q. acutissima* and *Q. sessilifolia*. Apically eaten leaves were found most frequently in *Q. sessilifolia*. In *Q. acutissima*, however, basally and centrally eaten leaves were found frequently. Leaf chemical components, particularly heterogeneous, microscale distributions of chemicals, may be related to these different manners of leaf‐eating. First, total phenolic content was higher at the margin of leaves than at the center in *Q. acutissima* which kept much higher phenolics levels than *Q. sessilifolia*. As suggested by Ito et al. (2016), *P. leucogenys* may avoid the leaf margin to avoid consuming the high concentrations of phenolics. However, they do not need to avoid the margin when eating *Q. sessilifolia* leaves because this tree leaves has lower, homogenously distributed phenolics. Second, sugar was distributed homogenously in both tree species, but *Q. acutissima* kept much higher sugar levels than *Q. sessilifolia*. Therefore, sugar is an important factor in the preference of *P. leucogenys* for *Q. acutissima* over *Q. sessilifolia* leaves, but it may not affect their feeding manners.

Other nonexclusive explanations of preference for leaf centers include avoidance of leaf margins that have spines and are relatively tough. Many plants employ structural defences, such as spines, hairs, or thickened leaves (reviewed by Hanley, Lamont, Fairbanks, & Rafferty, 2007; Farmer, 2014). Spines at the leaf margin are more developed in *Q. acutissima* than in *Q. sessilifolia* (Kitamura & Okamoto, 1981). Therefore, the possibility of eating the center to avoid spines will be examined in the future. Leaf toughness is another factor to consider with regard to mammal feeding manners. Toughness may be related to water content. According to our measurements, the leaves of both *Q. acutissima* and *Q. sessilifolia* contained less water at the leaf margin than at the center, although not so clear as in the latter. Thus, it is unlikely that *P. leucogenys* eats the central part of *Q. acutissima* leaves to avoid the lower water content at the margin, which likely makes it tougher to eat. Water contents, however, may explain why *P. leucogenys* often ate basally when eating *Q. acutissima* leaves, as there was more water in the basal parts of its leaves.

In conclusion, microscale distributions of chemicals within individual leaves revealed that phenolic concentrations (probably related to bitterness) and water contents (probably related to toughness) affect which parts they eat, but sugar concentration (probably related to sweetness) is an important contributor to which species of tree leaves they select as food.

## CONFLICT OF INTEREST

None declared.

## AUTHOR CONTRIBUTIONS

All authors conceived the ideas and designed methodology; MI and FH collected and analyzed the data; MI and FH led the writing of the manuscript. All authors contributed critically to the drafts and gave final approval for publication.
